# Racial and Ethnic Disparities in Health Status, Chronic Conditions, and Behavioral Risk Factors Among Prostate Cancer Survivors, United States, 2015

**DOI:** 10.5888/pcd18.200523

**Published:** 2021-04-22

**Authors:** Joëlle Atere-Roberts, Simone C. Gray, Ingrid J. Hall, Judith Lee Smith

**Affiliations:** 1 Epidemiology and Applied Research Branch, Division of Cancer Prevention and Control, Centers for Disease Control and Prevention, Atlanta, Georgia; 2 Department of Epidemiology, Gillings School of Global Public Health, University of North Carolina, Chapel Hill, North Carolina

## Abstract

**Introduction:**

Little is known about perceived health status and behavioral risk factors among prostate cancer survivors. The objective of this study was to describe racial and ethnic differences in self-reported health status, chronic conditions, and selected behavioral risk factors among prostate cancer survivors in the US.

**Methods:**

We used data from the 2015 National Health Interview Survey to calculate the prevalence of various levels of health status, chronic conditions, behavioral risk factors, and sociodemographic characteristics among prostate cancer survivors aged 50 years or older. We stratified results by race and ethnicity.

**Results:**

Of the 317 prostate cancer survivors in our sample, 33.1% reported no physical activity, 64.2% reported being current drinkers, 26.1% characterized their drinking as moderate/heavy, 42.3% reported being former smokers, and 8.7% were current smokers. Nearly one-third (29.1%) of survivors were obese (body mass index ≥30), and 15.1% had 3 to 6 chronic conditions. A greater percentage of White (29.7%) than Black (14.2%) or Hispanic (16.3%) survivors were moderate/heavy drinkers. A greater percentage of Black (16.2%) than White (7.5%) or Hispanic (7.3%) survivors were current smokers. A greater percentage of Black (25.1%) or Hispanic (27.7%) than White (11.4%) survivors had 3 to 6 chronic conditions.

**Conclusion:**

As the population of older men increases, prostate cancer diagnoses and those surviving the disease will also increase. Significant racial and ethnic group differences in behavioral risk factors and chronic conditions exist among prostate cancer survivors. Public health could prioritize efforts to improve health behaviors among prostate cancer survivors and use targeted interventions to address disparities.

**Summary**

**What is already known on this topic?**

Poor health status, behavioral risk factors, and chronic conditions can affect the health outcomes and quality of life of prostate cancer survivors in the US. Little is known about racial and ethnic disparities in these variables among prostate cancer survivors.

**What is added by this report?**

Significant racial and ethnic differences exist among prostate cancer survivors in alcohol use, smoking status, and physical activity. The prevalence of chronic conditions is greater among Black and Hispanic survivors than White survivors.

**What are the implications for public health practice?**

Public health efforts could prioritize coordinated, multilevel initiatives to assist prostate cancer survivors in addressing chronic conditions and behavioral risk factors and tailor interventions to selected survivor groups.

## Introduction

In 2017, an estimated 3.1 million men were living with a previous diagnosis of prostate cancer (prostate cancer survivors) in the US (1). This number is expected to increase because of the growth and aging of the US population (2). Despite the large number of prostate cancer survivors in the US, little is known about their perceived health status and behavioral risk factors. Smoking, physical inactivity, and weight gain can increase the risk of disease recurrence, progression, and/or death among men with prostate cancer (3). Furthermore, prostate cancer survivors may have other chronic conditions, such as diabetes, cardiovascular disease (heart disease, cerebrovascular disease, atherosclerosis, and aortic aneurysm/dissection), or hypertension, in addition to their cancer diagnosis (4,5). The prevalence of these chronic conditions is often higher among cancer survivors in racial and ethnic minority groups, such as Hispanic and non-Hispanic Black survivors, than among non-Hispanic White survivors (6). The management of multiple chronic conditions among prostate cancer survivors can affect the choice and uptake of cancer treatment, treatment outcomes, care delivery, and survivorship (7).

Poor health behaviors such as physical inactivity, cigarette smoking, and alcohol intake can increase the risk of recurrent and subsequent cancer diagnoses and other chronic conditions (8–11). Furthermore, the high prevalence of other chronic conditions in combination with the slow progression of a prostate tumor may result in prostate cancer survivors being more likely to die of other chronic conditions (12). Addressing the behavioral risk factors related to prevalent chronic conditions among prostate cancer survivors is imperative for public health intervention in this population. Improved health behaviors among all cancer survivors can enhance overall health status and lower the risk of recurrence and second primary cancers (13). Additionally, improved health behaviors may provide opportunities for better mental health and cognitive functioning, physical health outcomes, and overall quality of life in survivorship (14). The prevalence of chronic conditions among prostate cancer survivors has been examined; however, few studies have investigated racial and ethnic disparities in chronic health conditions that exist simultaneously (15). Identifying differences across race and ethnicity in self-reported health status, chronic conditions, and behavioral risk factors for this population could highlight the need for public health to prioritize the health needs of some racial and ethnic minority groups and inform research and practice activities aimed at enhancing health outcomes and quality of life of prostate cancer survivors.

To address gaps in the published literature, we used data from the 2015 National Health Interview Survey (NHIS) to describe self-reported health status, chronic conditions, and selected behavioral risk factors among prostate cancer survivors across race and ethnicity. No population-based studies have examined racial and ethnic differences in self-reported health status, behavioral risk factors, and multiple chronic conditions among prostate cancer survivors.

## Methods

The NHIS is an annual survey representative of the civilian, noninstitutionalized population aged 18 years or older residing in the US, administered by the National Center for Health Statistics at the Centers for Disease Control and Prevention (CDC) (www.cdc.gov/nchs/nhis/) (16). The NHIS collects data on demographic characteristics, health status, and health-related risk behaviors of adult respondents. The 2015 NHIS fielded the Cancer Control Supplement, which captures data on cancer-related behavioral risk factors, screening, and risk assessment. In 2015, the final response rate for the sample adult component was 55.7% (16). The study respondents were men who answered yes to the question “Have you ever been told by a doctor or other health professional that you had cancer or a malignancy of any kind?” and self-reported the cancer type as prostate. We restricted our analysis to men aged 50 years or older who reported a history of prostate cancer and no history of other cancers. We excluded men who reported any other cancer diagnosis. We analyzed 3 mutually exclusive race and ethnicity groups: non-Hispanic White (hereinafter, “White”), non-Hispanic Black or African American (hereinafter, “Black”), and Hispanic. We excluded Asian Americans and Pacific Islanders, American Indians, and Alaska Native populations because of insufficient sample sizes. Our final sample was 317 men.

We examined the following demographic characteristics: age (50–64, 65–74, or ≥75 y), marital status (married/living with a partner or divorced/widowed/separated/never married), education (<high school diploma, high school graduate/GED, some college, or college graduate), employment (yes or no), health insurance (public, private, or none), region (Northeast, Midwest, South, or West), and family history of prostate cancer (yes or no). We categorized a respondent who reported a father or brother(s) with a prostate cancer diagnosis as having a first-degree family history of prostate cancer.

Self-reported health was determined by response to the question “Would you say your health in general is excellent, very good, good, fair, or poor?” To ensure sufficient cell sizes when we stratified by race and ethnicity, we reclassified responses to a 3-level self-reported variable as follows: “excellent or very good,” “good,” and “fair or poor.” We also examined 3 behavioral risk factors: alcohol consumption, smoking status, and physical activity. We classified the variable for alcohol consumption as follows: never drinker, former drinker, infrequent/light current drinker, and moderate/heavy current drinker. We followed an approach used by the National Center for Health Statistics to classify alcohol consumption: former drinker (<12 drinks in any 1 year in lifetime but no drinks in the past year); infrequent drinking (1–11 drinks in the past year); light drinking (<3 drinks per week); moderate drinking (3–14 drinks per week); and heavy drinking (>14 drinks per week) (www.cdc.gov/nchs/nhis/alcohol/alcohol_glossary.htm). We collapsed the 6-level categorical variable for alcohol consumption into never, former drinker, current drinker (infrequent or light), and current drinker (moderate or heavy) to avoid small sample sizes caused by stratifying by race and ethnicity. We chose to separate current drinkers into infrequent/light and moderate/heavy to increase the level of detail in rates of alcohol consumption while maintaining adequate cell sizes. We used the National Center for Health Statistics recode for smoking status and reported smoking status as a 3-level variable: never, current (smoked 100 cigarettes in lifetime and currently smokes cigarettes), or former (previously smoked ≥100 cigarettes in lifetime but no longer currently smokes) (www.cdc.gov/nchs/nhis/tobacco/tobacco_glossary.htm). The physical activity variable was defined by using the 2008 US Department of Health and Human Services minimum physical activity recommendation (https://health.gov/our-work/physical-activity/previous-guidelines/2008-physical-activity-guidelines) of 150 minutes of moderate-intensity physical activity per week or 75 minutes of vigorous-intensity physical activity per week. We classified physical activity as a 3-level variable: no activity, some activity (<150 minutes of moderate-intensity physical activity per week or <75 minutes of vigorous-intensity activity per week), and met or exceeded (≥150 minutes of moderate-intensity physical activity per week or ≥75 minutes of vigorous-intensity activity per week).

We examined 6 chronic conditions: arthritis, asthma, heart disease, diabetes, hypertension, and obesity. We selected these conditions because they are amenable to behavioral intervention and had the highest prevalence among men aged 50 or older in the 2015 NHIS. Obesity was based on self-reported data for height and weight and defined as having a body mass index equal to or above 30 (measured as weight in kg divided by height in m^2^). We classified the remaining chronic conditions as yes or no in response to the question “Have you ever been told by a doctor or other health professional that you have [disease].” We quantified the total number of chronic conditions by using a 4-level variable to indicate the number of additional reported chronic conditions (0, 1, 2, and ≥3), similar to methods used previously (15). We defined having multiple chronic conditions as reporting of any 1 of the 6 conditions (arthritis, asthma, heart disease, diabetes, hypertension, or obesity) in addition to the prostate cancer diagnosis.

To account for the complex sampling design and to allow estimation of the national prevalence of the selected behavioral risk factors and chronic conditions, we weighted and analyzed all data estimates by using SAS version 9.4 (SAS Institute, Inc). We calculated overall weighted percentage estimates for demographic characteristics, selected behavioral risk factors, and chronic conditions, and we stratified data by race and ethnicity. We assessed overall differences across race and ethnicity using the Pearson χ^2^ test. For this analysis, *P* values less than .05 were considered significant.

## Results

In our sample of prostate cancer survivors aged 50 or older, 73.2% identified as White, 16.1% as Black, and 10.7% as Hispanic. Most were aged 65 or older (80.1%), were married or living with a partner (75.5%), were not employed (73.1%), had health insurance (98.2%), had some college or more (65.4%), and had no known family history of prostate cancer (75.5%) (Table 1). All sociodemographic characteristics varied significantly by race and ethnicity. The prevalence of the following characteristics was higher among White prostate cancer survivors than among Black and Hispanic survivors: being aged 65 or older, married, employed, college graduate, and having private health insurance. The prevalence of having less than a high school diploma was approximately 3 times higher among Hispanic men (32.6%) than among White men (10.5%) and Black men (11.8%). Hispanic men also had a higher prevalence of being uninsured (8.3%) than did White men (0.8%) and Black men (2.4%). Black men had a higher prevalence of not being employed (83.9%) than White men (70.7%) and Hispanic men (75.8%).

**Table 1:** Self-Reported Sociodemographic Characteristics of Men Aged ≥50 With a Previous Prostate Cancer Diagnosis, by Race and Ethnicity, United States, 2015^a^

Characteristic	Overall (N = 317)	Non-Hispanic White (n = 232)	Non-Hispanic Black (n = 51)	Hispanic (n = 34)	*P* Value^b^
**Age, y**					
50–64	19.9 (15.8–23.9)	16.7 (12.8–20.6)	30.2 (20.0–40.4)	28.0 (11.6–44.4)	.009
65–74	41.5 (37.5–45.5)	43.2 (38.2–48.2)	39.0 (31.0–47.0)	32.9 (21.7–44.0)	
≥75	38.6 (34.9–42.4)	40.0 (35.7–44.4)	30.8 (25.3–36.2)	39.2 (23.3–55.0)	
**Marital status**					
Married/living with partner	75.5 (72.3–78.8)	77.7 (74.2–81.1)	69.8 (63.2–76.3)	68.5 (54.8–82.2)	.049
Divorced/widowed/separated/single	24.5 (21.2–27.7)	22.3 (18.9–25.8)	30.2 (23.7–36.8)	31.5 (17.8–45.2)	
**Education**					
<High school diploma	13.1 (9.61–16.6)	10.5 (6.54–14.5)	11.8 (6.32–17.2)	32.6 (19.2–46.0)	.004
High school graduate/GED	21.5 (18.5–24.5)	19.6 (15.9–23.3)	27.4 (21.8–33.0)	27.1 (14.9–39.3)	
Some college	25.6 (22.3–28.8)	25.9 (22.2–30.0)	29.8 (23.7–35.9)	17.7 (9.59–25.9)	
College graduate	39.8 (35.2–44.4)	44.0 (38.6–49.3)	31.1 (21.7–40.5)	22.6 (9.15–36.0)	
**Employed**					
Yes	26.9 (22.7–31.2)	29.3 (23.9–34.8)	16.1 (13.8–18.4)	24.2 (10.3–38.1)	.001
No	73.1 (68.8–77.3)	70.7 (65.2–76.1)	83.9 (81.6–86.2)	75.8 (61.9–89.7)	
**Health insurance**					
Private	60.6 (57.3–63.9)	64.6 (60.2–68.9)	62.1 (53.0–71.2)	31.2 (17.5–44.9)	<.001
Public	37.6 (34.3–40.9)	34.7 (30.4–38.9)	35.4 (26.2–44.7)	60.5 (46.4–74.5)	
None	1.8 (1.3–2.4)	0.8 (0.1–1.5)	2.4 (2.1–2.7)	8.3 (6.6–10.0)	
**Region**					
Northeast	17.7 (16.5–18.9)	18.3 (17.1–19.5)	14.3 (12.4–16.2)	—c	<.001^d^
Midwest	21.7 (19.7–23.7)	22.4 (20.2–24.5)	18.1 (14.2–22.0)	—c	
South	42.0 (38.6–45.3)	38.8 (35.5–42.1)	58.5 (53.0–64.1)	—c	
West	18.7 (16.8–20.5)	20.5 (18.4–22.6)	9.05 (7.54–10.6)	—c	
**Family history of prostate cancer**					
Yes	24.5 (21.3–27.6)	24.0 (20.2–27.8)	30.2 (22.6–37.9)	20.2 (12.0–28.4)	.19
No	75.5 (72.4–78.7)	76.0 (72.2–79.8)	69.8 (62.1–77.4)	79.8 (71.5–88.0)	

a Source: 2015 National Health Interview Survey. All values are weighted percentage (95% CI).

b Pearson χ^2^ test used to generate *P* values.

c Sample size too small to report.

d χ^2^ test did not include Hispanic participants because of small sample size.

Overall, 43.2% of prostate cancer survivors reported excellent or very good self-reported health, and 24.4% reported fair or poor health (Table 2). Current alcohol consumption was reported by 64.2% of survivors; 38.1% reported infrequent or light drinking and 26.1% reporting moderate or heavy drinking. Current smoking was reported by 8.7% of survivors; 42.0% reported meeting or exceeding the minimum physical activity guidelines, 24.9% reported some physical activity, and 33.1% reported no physical activity.

**Table 2:** Self-Reported Health Status and Selected Behavioral Risk Factors Among Men Aged ≥50 With a Previous Prostate Cancer Diagnosis, by Race and Ethnicity, United States, 2015^a^

Characteristic	Overall (N = 317)	Non-Hispanic White (n = 232)	Non-Hispanic Black (n = 51)	Hispanic (n = 34)	*P* Value^b^
**Self-reported health**					
Excellent/very good	43.2 (38.9–47.4)	49.1 (44.1–54.1)	25.6 (16.5–34.7)	25.1 (11.6–38.7)	<.001
Good	32.4 (29.5–35.3)	29.9 (26.3–33.6)	39.3 (32.4–46.2)	40.9 (30.3–51.4)	
Fair/poor	24.4 (20.5–28.3)	21.0 (15.9–26.1)	35.1 (29.9–40.3)	34.0 (19.6–48.3)	
**Alcohol consumption**					
Never drinker	11.9 (8.6–15.2)	9.9 (6.0–13.7)	18.9 (15.6–22.1)	17.0 (2.9–31.1)	<.001
Former drinker	23.9 (19.5–28.3)	21.1 (16.1–26.0)	33.6 (24.7–42.6)	31.1 (17.8–44.4)	
Current drinker, infrequent/light	38.1 (33.5–42.7)	39.3 (33.8–44.9)	33.3 (24.4–42.2)	35.5 (25.0–46.0)	
Current drinker, moderate/heavy	26.1 (22.3–29.9)	29.7 (25.4–34.1)	14.2 (4.51–23.9)	16.3 (8.06–24.6)	
**Smoking status**					
Never smoker	49.0 (45.3–52.7)	50.4 (45.8–54.9)	35.7 (28.8–42.7)	57.1 (41.0–73.1)	.04
Current smoker	8.7 (6.6–10.8)	7.5 (5.9–9.1)	16.2 (7.77–24.6)	7.3 (0–18.0)	
Former smoker	42.3 (38.6–46.0)	42.1 (37.6–46.7)	48.1 (38.7–57.4)	35.6 (20.2–50.9)	
**Physical activity**					
No activity	33.1 (29.1–37.1)	29.8 (25.3–34.4)	43.1 (35.4–50.8)	42.5 (29.6–55.3)	.004
Some activity	24.9 (21.2–28.6)	26.8 (22.5–31.1)	15.1 (9.81–20.4)	24.3 (10.9–37.6)	
Meets/exceeds	42.0 (38.0–46.0)	43.3 (38.8–47.8)	41.8 (32.6–51.0)	33.2 (24.3–42.2)	

a Source: 2015 National Health Interview Survey. All values are weighted percentage (95% CI).

b Pearson χ^2^ test used to generate *P* values.

Self-reported health status and behavioral risk factors varied significantly overall by race and ethnicity (*P* < .05) (Table 2). The prevalence of excellent or very good self-reported health was higher among White men (49.1%) than Black men (25.6%) and Hispanic men (25.1%), whereas the prevalence of fair or poor self-reported health was higher among Black men (35.1%) and Hispanic men (34.0%) than among White men (21.0%). White men had a higher prevalence (29.7%) of moderate or heavy alcohol consumption among current drinkers than Black men (14.2%) and Hispanic men (16.3%). The prevalence of current smoking was higher among Black men (16.2%) than among White men (7.5%) and Hispanic men (7.3%). The prevalence of no physical activity was lower among White men (29.8%) than among Black men (43.1%) and Hispanic men (42.5%).

Overall, the chronic conditions with highest prevalence among prostate cancer survivors were arthritis (46.3%), hypertension (62.2%), and obesity (29.1%) (Table 3). We observed differences in the prevalence of the 6 chronic conditions by race and ethnicity. The prevalence of arthritis was lower among Hispanic men (25.6%) than among Black men (55.5%) and White men (47.5%). The prevalence of diabetes was higher among Hispanic men (46.5%) than among Black men (25.2%) and White men (10.2%). Hypertension and obesity were prevalent across the 3 groups, and we found significant differences by race and ethnicity. The prevalence of hypertension was higher among Black men (79.1%) than White men (58.0%) and Hispanic men (69.3%). A similar pattern emerged for obesity: 46.4% of Black men, 28.6% of Hispanic men, and 25.9% of White men had a body mass index of 30 or more.

**Table 3:** Self-Reported Chronic Conditions Among Men Aged ≥50 With a Previous Prostate Cancer Diagnosis, by Race and Ethnicity, United States, 2015^a^

Characteristic	Overall (N = 317)	Non-Hispanic White (n = 232)	Non-Hispanic Black (n = 51)	Hispanic (n = 34)	*P* Value^b^
**Arthritis**					
Yes	46.3 (42.3–50.3)	47.5 (42.6–52.5)	55.5 (47.4–63.6)	25.6 (18.8–32.4)	<.001
No	53.7 (49.7–57.7)	52.5 (47.5–57.4)	44.5 (36.4–52.6)	74.4 (67.5–81.2)	
**Asthma**					
Yes	7.52 (6.02–9.01)	8.39 (6.70–10.1)	6.91 (1.44–12.4)	2.25 (1.78–2.73)	<.001
No	92.5 (91.0–94.0)	91.6 (89.9–93.3)	93.1 (87.6–98.6)	97.7 (97.3–98.2)	
**Heart Disease**					
Yes	16.0 (12.2–19.8)	16.2 (11.7–20.7)	18.6 (14.0–23.3)	11.0 (0–24.1)	.49
No	84.0 (80.2–87.8)	83.8 (79.3–88.2)	81.4 (76.7–86.0)	89.0 (75.9–100.0)	
**Diabetes**					
Yes	16.3 (13.5–19.0)	10.2 (8.00–12.5)	25.2 (18.2–32.1)	46.5 (35.8–57.2)	<.001
No	83.7 (81.0–86.4)	89.8 (87.5–92.0)	74.8 (67.9–81.7)	53.5 (42.8–64.2)	
**Hypertension**					
Yes	62.2 (57.8–66.7)	58.0 (52.3–63.7)	79.1 (74.9–83.2)	69.3 (57.2–81.4)	<.001
No	37.8 (33.3–42.2)	42.0 (36.3–47.7)	20.9 (16.8–25.1)	30.7 (18.5–42.8)	
**Obesity (body mass index **≥**30.0 kg/m^2^)**					
Yes	29.1 (25.8–32.4)	25.9 (22.2–29.6)	46.4 (38.6–54.1)	28.6 (19.1–38.1)	<.001
No	70.9 (67.6–74.2)	74.1 (70.4–77.8)	53.6 (45.8–61.4)	71.4 (61.9–80.9)	
**Number of chronic conditions**					
0	23.1 (19.3–26.9)	25.5 (20.5–30.4)	9.1 (7.2–11.0)	25.0 (12.8–37.1)	<.001
1	39.1 (35.1–43.0)	42.5 (37.7–47.3)	35.4 (27.3–43.4)	19.9 (8.99–30.9)	
2	22.7 (19.2–26.2)	20.6 (17.2–23.9)	30.4 (25.9–35.0)	27.4 (9.81–45.0)	
3–6	15.1 (12.2–18.1)	11.4 (7.8–15.1)	25.1 (19.9–30.4)	27.7 (19.7–35.7)	

a Source: 2015 National Health Interview Survey. All values are weighted percentage (95% CI).

b Pearson χ^2^ test used to generate *P* values.

For the number of chronic conditions, 23.1% of men reported having no additional chronic conditions and 76.9% of men reported having 1 or more chronic conditions in addition to their cancer diagnosis (Table 3). The prevalence of having no chronic conditions was lowest among Black men (9.1%). Conversely, the prevalence of having 3 to 6 chronic conditions was higher among Black men (25.1%) and Hispanic men (27.7%) than White men (11.4%).

## Discussion

Using data from the 2015 NHIS, we found differences in the prevalence of selected behavioral risk factors, levels of self-reported health, and chronic conditions among White, Black, and Hispanic men with a previous prostate cancer diagnosis. Multiple chronic conditions were common among prostate cancer survivors in our sample; most had at least 1 chronic condition in addition to their cancer diagnosis ***.*** Further exploration is warranted to fully understand the effect of multiple chronic conditions among prostate cancer survivors to improve disease management and increase survival rates. Our findings are timely given the COVID-19 pandemic. Obesity and diabetes, chronic conditions examined in our study, are associated with poor COVID-19 outcomes (17). The prevalence of multiple chronic conditions in this population may put prostate cancer survivors at higher risk for emerging infectious diseases such as the novel coronavirus.

Previous studies documented racial and ethnic differences in behavioral risk factors and health status among survivors of multiple types of cancer (18,19). However, few studies examined racial and ethnic differences in behavioral risk factors and the number of chronic conditions among prostate cancer survivors (15). In a study of all cancer types, Nayak et al found that, compared with White survivors, Black survivors were less likely to meet physical activity guidelines, and Black and Hispanic survivors were more likely to be overweight or obese consistent with our findings among prostate cancer survivors. (19). Findings among prostate cancer survivors aged 40 or older in a study by Li et al found racial and ethnic differences in the total number of chronic conditions (15). Their study, which used data from the Behavioral Risk Factor Surveillance System, found that prostate cancer survivors who were Hispanic or in “other” racial/ethnic groups (Asian American, Native Hawaiian/Other Pacific Islander, American Indian/Alaska Native) had the highest prevalence of having 3 or more chronic conditions. Our study found a higher prevalence of 3 or more conditions among Hispanic and Black prostate cancer survivors than among White survivors. Other studies have shown greater prevalence of chronic disease among prostate cancer survivors than among the general population (14). Our findings highlight the burden of chronic diseases among prostate cancer survivors above and beyond the cancer diagnosis. An observational study that examined Surveillance, Epidemiology, and End Results (SEER) data from 1973–2012 found an increased risk of death from cardiovascular disease among survivors of several cancers, including prostate cancer (5).

Most prostate cancer survivors in our study reported current alcohol consumption. Although the evidence for the association between alcohol and prostate cancer is inconsistent, the International Agency for Research on Cancer has classified alcohol as a Group I carcinogen in humans (20). A recent population-based study in Canada found that higher prostate cancer–specific mortality was associated with postdiagnosis consumption of more than 2 alcoholic drinks per day compared with nondrinkers (9). Although studies of alcohol consumption among prostate cancer survivors are limited, counseling to minimize exposure to alcohol consumption after diagnosis (21,22) may be justified and may have implications for cancer care, treatment, and survivorship.

The prevalence of smoking in our sample of men was lower than that among men in the general US population (23). However, smoking is known to cause cancer, and cancer survivors who continue to smoke after diagnosis are at increased risk for recurrent and subsequent malignancies (10,24). Furthermore, continued smoking after a prostate cancer diagnosis is associated with increased all-cause and cancer-specific mortality (25). Our findings demonstrate the higher prevalence of current smoking among Black prostate cancer survivors, compared with White and Hispanic survivors, which suggests that interventions should be tailored to this population. Health care providers could reinforce messages about the increased risks associated with continued smoking after a prostate cancer diagnosis and provide referrals for evidence-based smoking cessation resources, including cessation counseling and medications approved for cessation support (26). Furthermore, additional efforts to improve health care system initiatives to reduce smoking among prostate cancer survivors, particularly Black prostate cancer survivors, may be warranted (27).

Current physical activity recommendations for adults are that adults engage in at least 150 minutes of moderate-intensity activity or 75 minutes of vigorous-intensity activity per week (28). More than one-third of prostate cancer survivors in our study reported no physical activity, with more than 42% of Black and Hispanic prostate cancer survivors each reporting no physical activity. Given the large proportion (38.6%) of survivors aged 75 or older, it is important to consider the psychological and cognitive benefits accruing from regular exercise among older men in racial and ethnic minority groups (11,28).

Many studies of prostate cancer survivors have reported on the outcomes associated with sexual function, urinary or bowel incontinence, or the emotional side effects after treatment (29,30). Our study contributes to a gap in research on the physical health and behavioral risk factors of prostate cancer survivors. Our findings suggest the need for research on whether prostate cancer survivors are receiving culturally appropriate prevention messages and social support for optimally managing behavioral risk factors and the challenges posed by multiple chronic conditions. The Community Guide recommends strategies to promote healthy behaviors and limit excessive alcohol use, physical inactivity, and smoking (https://www.thecommunityguide.org). We did not examine social determinants of health. However, our findings identify patterns in health status, chronic conditions, and health behaviors that may warrant a future examination of the social determinants of health and their effect on prostate cancer survivors. In addition, future research might seek to determine how social determinants of health affect preventive behaviors related to mental health, stress, and coping and prostate cancer survivors’ participation in positive health practices.

Our study had several limitations. First, survey data were self-reported and not confirmed by medical record review. Self-reported health status is commonly used as an outcome of interest; however, it is more subjective than other indicators of health status. Second, the cross-sectional study design did not allow examination of temporal relationships. Third, we did not assess whether chronic conditions were being managed effectively. Fourth, because we used 2015 data, our results may or may not represent the composition of current prostate cancer survivors. Finally, we did not have sufficient sample size in this single year of data to examine the variables of interest among Asian American, Pacific Islander, American Indian, and Alaska Native survivors. Because the objective of this study was to identify differences across race and ethnicity among prostate cancer survivors, we cannot draw inferences for racial and ethnic groups. Further research is needed to understand more about the differences highlighted in our findings.

After skin cancer, prostate cancer is the most common cancer diagnosed among US men. Most men with this disease survive at least 5 years, and thus, the projected increase in prostate cancer diagnoses, because of the aging of the US population, inevitably will lead to a greater number of survivors. We observed important differences in behavioral risk factors by race and ethnicity that could affect the health and well-being of the growing number of prostate cancer survivors. Our findings clearly present opportunities to improve behavioral risk factors among prostate cancer survivors. Public health efforts at multiple levels aimed at health promotion are imperative to improve overall health status and health-related quality of life, and potentially prevent a recurrence or a second primary cancer diagnosis. Our findings suggest that prostate cancer survivors may benefit from more education and consultation about ways to reduce behavioral risk factors and from community-based and health care system interventions to promote physical activity and reduce smoking and excessive alcohol consumption. Our results highlight the need to address racial and ethnic disparities among prostate cancer survivors by tailoring health and wellness services and interventions for racial and ethnic minority groups. Importantly, patient, provider, and system-level improvements, such as integrating follow-up care across providers and coordinated health promotion efforts to reduce behavioral risk factors, may be needed to help health care providers and patients address chronic health conditions and behavioral risk factors in a coordinated manner.
